# Supplementary material for: A prophage-encoded sRNA limits phage infection of adherent-invasive E. coli
Source: PLoS Pathog. 2026 Jan 2;22(1):e1013836. doi: 10.1371/journal.ppat.1013836 (PMC12803456; doi:10.1371/journal.ppat.1013836)
Supplement: S1 Text — Fig A. Comparative analysis of NC-SV and lambda prophages. Genomic alignment between the NC-SV prophage and phage Lambda. Arrows represent predicted open reading frames. Homologous open reading frames are connected by ribbons shaded in gray-scale according to pairwise amino-acid identity (0–100%). Fig B. RSB wastewater phage genome comparisons. Phages RSB01–04 were isolated from the headwaters of the wastewater treatment plant in Missoula, Montana, USA. Phage RSB01 (44,523 bp; NCBI accession PX402465) and RSB03 (43,192 bp; NCBI accession PX402467) are both Veterinaerplatzviruses that share 51.7% nucleotide homology, while phages RSB02 (76,472 bp; NCBI accession PX402466) and RSB04 (76,787 bp; NCBI accession PX402468) are related Kuraviruses that share 60.7% nucleotide homology. The genome comparisons shown here were generated by Clinker and homologous open reading frames are connected by ribbons shaded in gray-scale according to pairwise amino-acid identity (0–100%). Fig C. Phage titers of RSB01–RSB04 on NC101 and prophage-deletion mutants. E. coli NC101 and its isogenic prophage deletion derivatives (NC101ΔNC-SV, NC101ΔNC-MV, and NC101ΔNC-Ino) were infected with wastewater-derived phages RSB01–RSB04. Phage titers were quantified as plaque-forming units per milliliter (PFU/mL) 6 h post-infection. Data are the mean ± SEM from three independent experiments, *P < 0.05, determined by unpaired Student’s t test comparing PFUs on each mutant strain to wild-type NC101; ns, not significant. Fig D. The NC-SV prophage protects E. coli NC101 from infection by some, but not all phages by reducing virion adsorption. (A) Representative images of plaques formed by wastewater phage isolates RSB01, RSB02, or RSB04 on WT NC101 or the indicated prophage mutants are shown. (B-D) The surface area of plaques formed on lawns of the indicated strains was measured, N = 50 plaques per condition, from four replicate experiments, ****P < 0.0001; ns = not significant. (E-G) Growth of WT NC101 an [file ppat.1013836.s001.docx]

**Supporting Information for: A prophage-encoded sRNA**

**limits phage infection of adherent-invasive *E. coli***

Nicole L. Pershing^1#^, Robert S. Brzozowski^2#^, Amelia K. Schmidt^2#^, Annika Dankwardt^1^,

Dominick R. Faith^4^, Alex C. Joyce^4^, Lizett Ortiz de Ora^4^, John D. Kominsky^4^, Andrew Maciver^2^, Rickesha Bell^3^, William S. Henriques^4^, Shelby E. Andersen^5^, Blake Wiedenheft^4^, Sherwood R. Casjens^3^, Breck A. Duerkop^5^, June L. Round^3, *^, and Patrick R. Secor^4, 6, *^

Affiliations:

^1^Department of Pediatrics, Division of Pediatric Infectious Diseases, University of Utah School of Medicine, Salt Lake City, Utah, USA

^2^Division of Biological Sciences, University of Montana, Missoula, MT, USA

^3^Department of Pathology, University of Utah School of Medicine, Huntsman Cancer Institute, Division of Microbiology and Immunology, Salt Lake City, UT, USA

^4^Department of Microbiology & Cell Biology, Montana State University, Bozeman, MT, USA

^5^Department of Immunology and Microbiology, School of Medicine, University of Colorado – Anschutz Medical Campus, School of Medicine, Denver CO, USA

^6^Center for Biofilm Engineering, Montana State University, Bozeman, MT, USA

^#^Equal contributions

*Correspondence: [june.round@path.utah.edu](mailto:june.round@path.utah.edu) and [patrick.secor@montana.edu](mailto:patrick.secor@montana.edu)


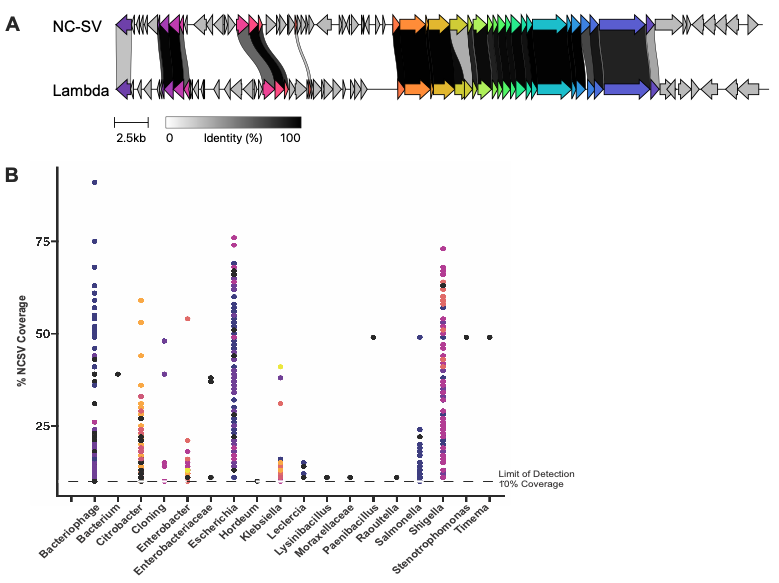


**Figure A. Comparative analysis of NC-SV and lambda prophages.**Genomic alignment between the NC-SV prophage and phage Lambda. Arrows represent predicted open reading frames. Homologous open reading frames are connected by ribbons shaded in gray-scale according to pairwise amino-acid identity (0–100%).

**Figure B. RSB wastewater phage genome comparisons.** Phages RSB01–04 were isolated from the headwaters of the wastewater treatment plant in Missoula, Montana, USA. Phage RSB01 (44,523 bp; NCBI accession PX402465) and RSB03 (43,192 bp; NCBI accession PX402467) are both Veterinaerplatzviruses that share 51.7% nucleotide homology, while phages RSB02 (76,472 bp; NCBI accession PX402466) and RSB04 (76,787 bp; NCBI accession PX402468) are related Kuraviruses that share 60.7% nucleotide homology. The genome comparisons shown here were generated by Clinker and homologous open reading frames are connected by ribbons shaded in gray-scale according to pairwise amino-acid identity (0–100%).

**Figure C. Phage titers of RSB01–RSB04 on NC101 and prophage-deletion mutants.**

*E. coli* NC101 and its isogenic prophage deletion derivatives (NC101^ΔNC-SV^, NC101^ΔNC-MV^, and NC101^ΔNC-Ino^) were infected with wastewater-derived lytic phages RSB01–RSB04. Phage titers were quantified as plaque-forming units per milliliter (PFU/mL). Data are the mean ± SEM from three independent experiments, *P<0.05, determined by unpaired Student’s t test comparing PFUs on each mutant strain to wild-type NC101; ns, not significant.

**Figure D. The NC-SV prophage protects *E. coli* NC101 from infection by some, but not all phages by reducing virion adsorption. (A)** Representative images of plaques formed by wastewater phage isolates RSB01, RSB02, or RSB04 on WT NC101 or the indicated prophage mutants are shown. **(B-D)** The surface area of plaques formed on lawns of the indicated strains was measured, N=50 plaques per condition, from four replicate experiments, ****P<0.0001; ns = not significant. **(E-G)** Growth of WT NC101 and NC101^ΔNC-SV^ was measured after infection with the indicated phages at an MOI of one. **(H-J)** The percentage of adsorbed virions was measured in WT NC101 or NC101^ΔNC-SV^ cells at the indicated times post infection, MOI=1. Data are the mean ± SEM of three experiments, ***P<0.001.

**Figure E. The NC-SV prophage is dispensable for intestinal colonization of mice without baseline E. coli colonization. (A–B)** Specific pathogen-free (SPF) C57BL/6J mice were screened to confirm lack of baseline Enterobacteriaceae from fecal pellets, then stably colonized with the indicated NC101 E. coli strains via daily oral gavage (10^8^ CFU, days 0–4 indicated by vertical dashed lines, n=10 female mice/group). **(A)** Quantification of NC101 colony forming units (log_10_ CFU/g feces, mean ± 95% CI) from fecal pellets plated on selective LB-chloramphenicol agar. P=0.2725, mixed-effects model with Geisser-Greenhouse correction. **(B)** Detectable log_10_ CFU mean ±- 95% CI for the indicated specimens collected after 2–3 weeks of colonization. Significantly different groups are indicated by corresponding letter design, where uppercase letters above each bar denote significantly different (P<0.05) groups by two-way ANOVA and post hoc means testing (Tukey) comparing all groups. Groups that share a letter designation are not statistically different (e.g. A is significantly different from B, but not AB). **(C–D)** The NC-SV prophage confers intestinal colonization fitness in the setting of baseline E. coli colonization. C57BL/6J PhyLo11B gnotobiotic mice stably colonized with a defined consortia of 11 bacteria (including the E. coli strain Mt1B1) were gavaged with the indicated NC101 strains daily via oral gavage (10^8 CFU, days 0–4 indicated by vertical dashed lines, n=15 mice/group, mixed male and female mice). (C) NC-SV deletion impairs competitive intestinal colonization. Quantification of NC101 colony forming units (log10 CFU/g feces, mean ± 95% CI, yellow and orange lines) from fecal pellets plated on selective LB-chloramphenicol agar compared to total *E. coli* CFU quantified on MacConkey Agar (representing Mt1B1 and NC101, magenta and pink lines). ****P<0.0001, ns, not significant; mixed-effects model with Geisser-Greenhouse correction. (D) Loss of detectable NC101 is hastened in the strain lacking the NC-SV prophage in Phy01t18 gnotobiotic mice with high baseline E. coli colonization. The percentage of mice with consistent detectable NC101 from selective plating of feces on LB-chloramphenicol agar is plotted by for each strain. **P<0.01, Mantel-Cox proportional hazard test.**

**Figure F. Comparative genome maps of E. coli NC101 and Mt1B1. Chromosomes of NC101 and Mt1B1 were annotated using Prokka. The two strains share 99.99% average nucleotide identity, and both encode the three NC101 prophages described in this study. Gene organization and prophage positions are highly conserved between the strains, underscoring their close genetic relatedness.**

**Figure G. The NC-SV prophage is dispensable for intestinal monocolonization of gnotobiotic mice.** The NC-SV prophage does not affect intestinal colonization or extraintestinal dissemination in gnotobiotic mice monocolonized with NC101. 129Sv/Ev germ-free (GF) mice were colonized with NC101 (n=8) or NC101^ΔNC-SV^ (n=8) E. coli via single oral gavage at 10-weeks of age (10^8^ CFU, day 0).
**(A)** Weight gain normalized to starting weight. P=0.13, mixed-effects model with Geisser-Greenhouse correction. **(B)** Quantification of NC101 colony forming units (log10 CFU/g feces, mean +/- 95% CI) from fecal pellets plated on selective LB-chloramphenicol agar. P=0.54, mixed-effects model with Geisser-Greenhouse correction. **(C)** Detectable log10 CFU (mean +/- 95% CI) for the indicated specimens collected 4 months following initial colonization. Significantly different groups are indicated by compact letter display, where uppercase letters above each bar denote significantly different (P<0.05) groups by two-way ANOVA and post hoc means testing (Tukey) comparing all groups. Groups that share a letter designation are not statistically different (e.g., A is significantly different from B, but not AB). SI, small intestine. CFU, colony forming units.

**Figure H. Phage RSB03 replicates in the mouse gut.** **(A)** Schematic overview of the experimental design created with BioRender.com. Specific pathogen-free (SPF) C57BL/6J mice were stably colonized with NC101 *E. coli* followed by oral administration of either active or heat-inactivated RSB03 phage (3x10^7^ PFU per dose, 2 doses, 7-hours apart). Created in BioRender. Bell, R. (2026) https://BioRender.com/jg98yai. **(B)** Quantification of *E. coli* colony forming units (log_10_ CFU/g feces, mean +/- 95% CI) from fecal pellets plated on selective LB-chloramphenicol agar; there were no statistically significant differences between strains. **(C)** Quantification of phage plaque-forming units (log_10_ PFU/g feces, mean +/- 95% CI) from fecal pellets; mice that received active RSB03 had significantly higher detectable PFU (P<0.0001, mixed-effects model with Geisser-Greenhouse correction) which was transiently detected the day of RSB03 administration and up to 12 days after administration.

**Figure I. The NC-SV prophage is associated with reduced *E. coli* dissemination to extraintestinal tissues and lower phage loads in the gut of monoassociated gnotobiotic mice. (A)** Schematic overview of experimental design. Germ-free C57BL/6J mice were stably colonized with either NC101 or NC101^∆NC-SV^ *E. coli*, then administered 1x10^8^ PFU RSB03 daily by gavage for five consecutive days. Fecal pellets, luminal contents, and tissues were collected for quantification of colony-forming units (CFU) and plaque forming units (PFU). Created in BioRender. Bell, R. (2026) https://BioRender.com/r8m130m. (**B-D**) Detectable *E. coli* CFU (**B**), RSB03 PFU (**C**) and Phage:Host index (**D**, min-max normalized log difference of PFU-CFU, with 1 reflecting equal density) for the indicated samples at endpoint. SI, small intestine. For bar plots, dots represent individual subjects, bars represent the mean ± 95% confidence interval. Significantly different groups are indicated by compact letter display, where uppercase letters above each bar denote significantly different (P<0.05) groups by two-way ANOVA and post hoc means testing (Tukey) comparing all groups. Groups that share a letter designation are not statistically different (e.g. A is significantly different from B, but not AB).

**Figure J. Comparison of LamB sequences and structures from *E. coli* NC101 and *E. coli* K-12 (1MAL). (A)**LamB protein sequences were aligned using Clustal. Amino-acid differences between the two sequences are highlighted in red. The N-terminal signal peptide and periplasmic residues (1–25) shown in gray were omitted from the solved LamB crystal structure (PDB 1MAL). **(B)** Predicted LamB trimers from *E. coli* NC101 (AlphaFold model, orange) and the experimentally determined LamB structure (PDB 1MAL, green) are displayed as surface renderings. Residues that differ between NC101 and 1MAL LamB are shown in hot pink.

**Figure K. The impact of glucose and maltodextrin on phage virion adsorption to *E. coli* NC101.** The percentage of adsorbed RSB03 virions were measured at the indicated times post infection with an initial MOI of one. Data are the mean ± SEM of three experiments.

**Figure L. Distribution of *E. coli* genomes carrying NC-SV–like prophages by source and pathotype. (A)** Source categories of *E. coli* assemblies containing NC-SV–like prophages, classified from NCBI BioSample metadata. Clinical isolates were the largest defined group (n = 1,603), followed by environmental (n = 328) and laboratory reference strains (n = 109). A substantial fraction (n = 2,137) lacked sufficient metadata for source assignment (*Unknown*). **(B)** Pathotypes of *E. coli* genomes with NC-SV–like prophages identified by BLASTn (≥ 10% genome coverage) across 4,114 *E. coli* assemblies. Most strains were unassigned (n = 3,515), but NC-SV–like prophages were also detected among multiple pathogenic subgroups, including STEC/EHEC (n = 297), ETEC (n = 147), UPEC/ExPEC (n = 113), EPEC (n = 4), as well as laboratory K-12 lineage strains (n = 99) and the AIEC model strain NC101 (n = 2).
