# Supplementary material for: A prophage-encoded sRNA limits phage infection of adherent-invasive E. coli
Source: PLoS Pathog. 2026 Jan 2;22(1):e1013836. doi: 10.1371/journal.ppat.1013836 (PMC12803456; doi:10.1371/journal.ppat.1013836)
Supplement: Attachment — Submitted filename: Review of Brzozowski et al prophage sRNA.docx [file ppat.1013836.s004.docx]

Major comments

1. Because the NC-SV prophage could encode virulence or transmission factors, it is premature to conclude that sensitivity to predation by RSB03 is why the NC-SV deletion strain has a different bacterial load and dissemination in mice compared to the parent strain NC101. You must compare loads and dissemination for these two strains in mice in the absence of RSB03 phage.
2. The paper seems incomplete without knowing how the NC-SV-encoded sRNA downregulates LamB. Did you computationally search for putative targets of the sRNA?
3. Although there are differences in plaque area, there is no *in vitro* data regarding phage EOP? Wouldn’t you expect a change in EOP due to change in surface receptor expression?

Minor comments

1. Line 100: Where is the key for the color-coded functional modules? Same for Fig S2.
2. Line 122: Better to call them virulent phages throughout rather than lytic phages. Temperate phages have a lytic cycle, after all.
3. Figure 1:
A. Is ΔNC-MV plaque size also a bit smaller?
B. Panel D: move legend to side as it appears to be data points.
C. Max adsorption is reached by 10 min, so how much does this

difference matter at 5 min?

D. Panel E: It is unclear what is being shown here. Is the final panel showing a cell with a burst region of the cell envelope? The figure legend should say something to help the reader.
E. The conclusion that the cell surface receptor makes them more prone to lysis is one possibility. Couldn’t it also be that NC-SV encodes for a protein that triggers phage defense systems that recognize RSB01/03? If the cell surface receptor plays a role in membrane integrity, shouldn’t you show this through other methods such permeability to ethidium bromide or the like?
4. Figure 2:
A. The quality of the figure is bad. Too pixelated.
B. Panels G-I: there is no description in the legend to indicated what A, B, C or D mean above each bar graph (same comment for figure 5S).
5. Figure S6: Should you consider that inability of lambda phage to infect NC101 might also be due to phage super immunity that’s not related to the phage receptor, but suppression of phage cycle?
6. Figure S7: Why isn’t RSB01 adsorption being impacted by glucose supplementation? Does it have an alternative receptor?
7. Figure 6A: there’s a pretty big difference growthwise in empty vector vs svsR expressing cells. Why is this?
8. Figure 6: Although visually it appears svsR insertion behaves pretty much as WT from prior figures, it would be beneficial to compare it to WT NC101 to show if expression of svsR is sufficient to restore WT plaque area reduction and decrease in phage adsorption.
9. Material and methods: For the plaque assay, you don’t say how many cells were used or if they are in top agar, etc. Instead there is information regarding the mouse experiment.
