# Supplementary material for: A prophage-encoded sRNA limits phage infection of adherent-invasive E. coli
Source: PLoS Pathog. 2026 Jan 2;22(1):e1013836. doi: 10.1371/journal.ppat.1013836 (PMC12803456; doi:10.1371/journal.ppat.1013836)
Supplement: Attachment — Submitted filename: Response to Reviewer Comments.pdf [file ppat.1013836.s006.pdf]

We thank the Editor and Reviewers for their thoughtful and constructive evaluation of our manuscript. In response, we have substantially revised the paper to strengthen the evidence for how the NC-SV prophage limits phage infection in *E. coli* NC101. Major changes include new mouse colonization experiments comparing NC101 and the ΔNC-SV mutant in the absence of phage or in the presence of a competing strain of *E. coli* (Mt1B1). We also include results from new experiments with a *lamB* deletion mutant that indicate LamB is not the primary receptor for phage RSB03 but contributes to phage plaque expansion, suggesting that the NC-SV prophage alters additional features of the bacterial cell surface to restrict phage spread. We also improved figure quality and legends, incorporated new LamB structural comparisons (referencing PDB 1MAL), and added methodological details for mouse models, bacterial quantification, and data availability. Together, these revisions address all reviewer comments and clarify the broader significance of prophage-encoded regulatory RNAs in controlling phage–bacteria interactions.

Journal Requirements:

1) Please ensure that the CRediT author contributions listed for every co-author are completed accurately and in full.
At this stage, the following Authors/Authors require contributions: Robert S. Brzozowski, Amelia K. Schmidt, Nicole L. Pershing, Annika Dankwardt, Dominick R. Faith, Alex C. Joyce, Andrew Maciver, William S. Henriques, Shelby E. Andersen, Blake Wiedenheft, Sherwood R. Casjens, Breck A. Duerkop, June L. Round, and Patrick Secor. Please ensure that the full contributions of each author are acknowledged in the "Add/Edit/Remove Authors" section of our submission form.
The list of CRediT author contributions may be found here: https://journals.plos.org/plospathogens/s/authorship#loc-author-contributions

CRediT author contributions are now listed for all authors.

2) We ask that a manuscript source file is provided at Revision. Please upload your manuscript file as a .doc, .docx, .rtf or .tex. If you are providing a .tex file, please upload it under the item type u2018LaTeX Source Fileu2019 and leave your .pdf version as the item type u2018Manuscriptu2019.

A word doc was provided.

3) Please provide an Author Summary. This should appear in your manuscript between the Abstract (if applicable) and the Introduction, and should be 150-200 words long. The aim should be to make your findings accessible to a wide audience that includes both scientists and non-scientists. Sample summaries can be found on our website under Submission Guidelines:
https://journals.plos.org/plospathogens/s/submission-guidelines#loc-parts-of-a-submission

An author summary was provided.

4) We do not publish any copyright or trademark symbols that usually accompany proprietary names, eg ©, ®, or TM (e.g. next to drug or reagent names). Therefore please remove all instances of trademark/copyright symbols throughout the text, including:
- ® on page: 20
- TM on page: 20.

These symbols were removed.

5) We notice that your supplementary Figures are included in the manuscript file. Please remove them and upload them with the file type 'Supporting Information'. Please ensure that each Supporting Information file has a legend listed in the manuscript after the references list.

A separate Supporting Information file was provided and all Figure Legends are now at the end of the manuscript.

6) Some material included in your submission may be copyrighted. According to PLOSu2019s copyright policy, authors who use figures or other material (e.g., graphics, clipart, maps) from another author or copyright holder must demonstrate or obtain permission to publish this material under the Creative Commons Attribution 4.0 International (CC BY 4.0) License used by PLOS journals. Please closely review the details of PLOSu2019s copyright requirements here: PLOS Licenses and Copyright. If you need to request permissions from a copyright holder, you may use PLOS's Copyright Content Permission form. Please respond directly to this email and provide any known details concerning your material's license terms and permissions required for reuse, even if you have not yet obtained copyright permissions or are unsure of your material's copyright compatibility. Once you have responded and addressed all other outstanding technical requirements, you may resubmit your manuscript within Editorial Manager.
Potential Copyright Issues:
i) Figures S4A, and S5A. Please confirm whether you drew the images / clip-art within the figure panels by hand. If you did not draw the images, please provide (a) a link to the source of the images or icons and their license / terms of use; or (b) written permission from the copyright holder to publish the images or icons under our CC BY 4.0 license. Alternatively, you may replace the images with open source alternatives. See these open source resources you may use to replace images / clip-art:
- https://commons.wikimedia.org
- https://openclipart.org/.

These images were created with BioRender and the language "Created with BioRender.com" is now included where appropriate. We also included BioRender publication licenses for these figures with the resubmission.

7) Thank you for stating 'Raw sequencing reads have been deposited as part of BioProject PRJNA1225266 in the NCBI SRA database.' Please note that, though access restrictions are acceptable now, your entire minimal dataset will need to be made freely accessible if your manuscript is accepted for publication. This policy applies to all data except where public deposition would breach compliance with the protocol approved by your research ethics board. If you are unable to adhere to our open data policy, please kindly revise your statement to explain your reasoning and we will seek the editor's input on an exemption.

The entire dataset will be made public upon acceptance for publication.

8) Please amend your detailed Financial Disclosure statement. This is published with the article. It must therefore be completed in full sentences and contain the exact wording you wish to be published.
1) State the initials, alongside each funding source, of each author to receive each grant. For example: "This work was supported by the National Institutes of Health (####### to AM; ###### to CJ) and the National Science Foundation (###### to AM)."
2) State what role the funders took in the study. If the funders had no role in your study, please state: "The funders had no role in study design, data collection and analysis, decision to publish, or preparation of the manuscript."
3) If any authors received a salary from any of your funders, please state which authors and which

funders..
If you did not receive any funding for this study, please simply state: u201cThe authors received no specific funding for this work.u201d

Our financial disclosure statement was updated to read:

This work was supported by NIH grant R01DK124317 to J.L.R and P.R.S. P.R.S. was also supported by NIH grant P20GM103474. D.R.F. was supported by the NSF GRFP (366502). N.L.P was supported by the National Center for Advancing Translational Sciences of the National Institutes of Health under Award Numbers UM1TR004409 and K12TR004413 and a pilot grant from the Intermountain Foundation at Primary Children's Hospital. The content is solely the responsibility of the authors and does not necessarily represent the official views of the National Institutes of Health or other funding sources. The funders had no role in study design, data collection and analysis, decision to publish, or preparation of the manuscript.

Reviewers' Comments:

Part I - Summary
Please use this section to discuss strengths/weaknesses of study, novelty/significance, general execution and scholarship.

Reviewer #1: They find that a lambdoid prophage confers protection against virulent phages. This is an important finding and worthy of PloS Pathogens. The manuscript is very well written. However, more data are needed to support their findings. Also, they lack a mechanism for how the sRNA is reducing virulent phage receptor expression. Papers that have sRNAs as the central player, should report on what their relevant target(s) are, in my opinion.

Reviewer #2: The manuscript by Brzozowski et al reports the discovery and characterization of a very interesting interaction between E. coli prophage and lytic phage through modulation of metabolism. The manuscript employs a satisfying mix of in vitro and in vivo (mouse) experiments, as well as RNA sequencing. In general, I like this study and found the manuscript to be well written and on a topic that is broadly relevant. I have some relatively minor comments listed below, the most important of which relates to the apparent use of parametric statistical tests in contexts (mouse data) where non parametric tests are likely more appropriate.

The authors should ensure that the statistical test used is stated in each figure legend (throughout). There is a stats section in the methods but specific information should additionally be in each figure legend. The compact letter display for statistical differences is not clear which comparisons are made and which are significant (e.g. Fig 2).

We thank the reviewer for this helpful suggestion. We have revised all figure legends to explicitly state the statistical test(s) used. In addition, we clarified the compact letter display by specifying the comparisons represented and highlighting which groups are significantly different. The updated figure legends and revised figures now reflect these changes (see Fig 2 and others as noted).

In some cases (CFU and PFU etc data from mouse experiments like Fig 2 and S5 etc) the data do not look normally distributed, yet the methods state that the statistical tests used were t tests (parametric). It is strongly recommended to employ non parametric tests especially when the sample size is small (as

here) and the assumptions of normal distribution cannot be formally assessed as they could properly be if sample sizes were very large.

We appreciate the reviewer's careful attention to this point. For all CFU and PFU data from mouse experiments, we have reanalyzed the data using nonparametric Mann–Whitney tests to compare ranks between strains. The figure legends and Methods section have been updated accordingly (see Fig. 2 and Supplemental Fig. S8, which corresponds to Fig. S5 in the previous version of the manuscript).

Do the C57Bl/6 mice used (Fig S4A-B) have native E. coli in their microbiota?

No. The experiments were performed with C57BL/6J mice obtained from Jackson Laboratory, which characteristically lack baseline Enterobacteriaceae colonization. To confirm this, fecal samples from all mice were screened on MacConkey agar prior to colonization, and no Enterobacteriaceae were detected. This information has been included in the revised manuscript (Methods section and in Fig. S5).

It seems from the methods that the CFU quantification involves selection on chloramphenicol agar plates for NC101(line 604 in methods) but I recommend specifying in the figure legend both for thoroughness and because if selective plating was not performed then native E. coli could have affected counts by adding to the E. coli load present. Regardless, it seems interesting to know if E. coli are present in the mouse gut prior to NC101 colonization since it could provide interesting ecological context for the study. Along these lines, profiling of the microbiota (e.g. 16S amplicon sequencing) could provide information about if the e coli colonization and/or phage treatment altered microbiome composition.

We have added this information to the relevant figure legends for clarity. In addition, fecal samples from all mice were screened on MacConkey agar prior to colonization to confirm the absence of baseline Enterobacteriaceae. These details are now explicitly stated in the Methods section of the revised manuscript (see also our response above).

Figure 2 legend should indicate that mice are gnotobiotic (ex-germfree).
There is ~6 log higher E coli load in the gnotobiotic (ex germfree) mice (fig 2) compared with the SPF mice (Fig S4), with corresponding higher phage burden. Are the observed findings about extraintestinal dissemination and phage loads relevant when only they are only observed in a non-physiological context of E coli mono-colonization of gnotobiotic mice?

We thank the reviewer for these important comments. We have revised the Fig. 2 legend to specify that the mice were gnotobiotic (ex-germfree).

As the reviewer notes, intestinal NC101 burdens are higher in gnotobiotic monoassociated mice compared with SPF mice, consistent with prior studies [PMID: 154474, 159260, 20421387]. Use of gnotobiotic animals with defined microbiota allows detailed analysis of phage–bacteria interactions and concurrent quantification of bacterial invasiveness following colonization and lytic phage treatment, a context relevant to invasive *E. coli* infections after bacterial translocation from the gut [PMID: 23649557, 32213075]. Because extraintestinal dissemination in healthy SPF mice was rare, the gnotobiotic model was necessary to enable quantification of these interactions.

In response to the reviewer's specific concerns:

- To address whether baseline *E. coli* could confound colonization results or CFU counts, we performed a new **competitive colonization experiment (Fig. S5C–D)** in gnotobiotic mice co-colonized with NC101 and *E. coli*Mt1B1. Mt1B1 is an AIEC strain with 99.99% nucleotide identity to NC101 and has been used extensively to study colonization resistance. Colonization was quantified by selective LB–chloramphenicol agar for NC101 and MacConkey agar for total E. coli density. These data show that the NC-SV prophage confers a measurable fitness advantage for NC101 in the presence of a closely related *E. coli* competitor.
- To address the concern that dissemination and phage loads might only occur in the "non-physiological context" of monoassociation, we added new data in **Fig. S7A–C**, showing that extraintestinal CFU are routinely detectable in gnotobiotic monoassociated mice with no diffences between the NC101 and NC101ΔNC-SV strains. The gnotobiotic system permited quantitative assessment of phage treatment effects under conditions of high *E. coli* burden and dysbiosis, which is relevant to invasive human disease that would not have been possible in SPF settings which confer protection to extraintestinal dissemination.

Together, these additional data demonstrate that while extraintestinal dissemination of NC101 is rarely detected in healthy SPF mice (Fig. S4A–B), it is routinely detected in gnotobiotic mice (Fig. S6C), and that the NC-SV prophage provides a fitness advantage in the presence of closely related *E. coli* strains (Fig. S4C–D). These differences between WT and ΔSV strains *in vivo* are now explicitly shown in **Figs. S5 and S7**.

These details have been added to the **Results section, lines 175–198**, of the revised manuscript.

Do the gnotobiotic mice experience weight loss over the experiment?

We did not perform serial weight measurements in the gnotobiotic lytic phage study to minimize the risk of cross-contamination between cohorts. However, there were no gross changes in weight or signs of systemic illness associated with NC101 monoassociation or lytic phage treatment in the gnotobiotic cohort (Fig. 2). In addition, new Fig. S7 shows that gnotobiotic mice monoassociated with NC101 or NC101$^{\Delta NC\text{-}SV}$ did not exhibit strain-specific differences in weight, consistent with our prior observations in lytic phage treated NC101-monoassociated mice. These details have been added to the Results section (lines 188–198) of the revised manuscript.

Dissemination to extraintestinal tissues seems likely to be accompanied by inflammation and this seems relevant.

We agree that inflammation is an important aspect of extraintestinal dissemination. Prior work has shown that NC101 colonization of wild-type mice is not associated with significant intestinal inflammation or mesenteric CD4+ T cell–driven IL-12/23 cytokine production [PMID: 30576451]. In contrast, extraintestinal dissemination of other E. coli strains in germ-free mice has been linked to increased serum TNF-α [PMID: 20421387], and models of E. coli liver abscess [PMID: 38096412], peritonitis [PMID: 11342656], and sepsis [PMID: 36643320] are all associated with elevated serum cytokines. In addition, phages themselves have been reported to alter immune and cytokine responses [PMID: 37932118].

Although these inflammatory outcomes are of considerable interest, assessing cytokine responses to E. coli intestinal colonization and extraintestinal dissemination, and how these responses may be modulated by lytic phage treatment, is beyond the scope of the present study. The current study is

focused specifically on defining the role of the NC-SV prophage in NC101 colonization dynamics, extraintestinal dissemination, and susceptibility to lytic phage predation.

For Clinker analysis, the coloring indicates common cluster membership of genes based on global sequence alignments, not functional modules as stated in Fig S1 legend. The authors should include clearly stated NCBI accession numbers in Methods or in a supplementary table.

This change was made. Additionally, phage genomes have been submitted to NCBI and accession numbers are now listed in Figure S2: RSB01 PX402465, RSB02 PX402466, RSB03 PX402467, RSB04 PX402468.

For plaque assays (e.g. Fig 1, S3), the authors should make clear if biological replicates were performed.

Thank you for the suggestion. We now indicate that four biological replicates were used for the plaque assays in Figs 1 and three replicates in Fig S3.

I am quite skeptical of the statement and references cited of glucose/maltodextrin concentrations in the intestinal lumen (line 289). 40mM seems quite high – most glucose is absorbed in the small intestine and I would expect glucose concentration in the colon lumen to be vanishingly low. Furthermore, dietary changes would likely alter the concentration significantly so I am wary that this 40mM value is accurate. Instead, I suggest that the author perform plaque experiments using a varied range of concentrations (perhaps 10 fold) to assess the question of glucose/maltodextrin levels on LamB expression and plaque size.

We thank the reviewer for raising this valid point. We agree that glucose concentrations in the intestinal lumen are variable and generally lower than the 40 mM value cited previously. In response, we performed the suggested experiment testing a range of glucose and maltodextrin concentrations spanning three orders of magnitude (0.4 mM, 4 mM, and 40 mM). As shown in the revised Figure 4A, glucose supplementation reduced RSB03 plaque size in a concentration-dependent manner, whereas maltodextrin supplementation enhanced plaque size relative to both unsupplemented and glucose-treated conditions. These data confirm that carbohydrate availability influences RSB03 infection dynamics in a graded fashion. We have updated the Results and Discussion to reflect these findings and revised the text to avoid implying that 40 mM glucose accurately represents physiological concentrations in the gut.

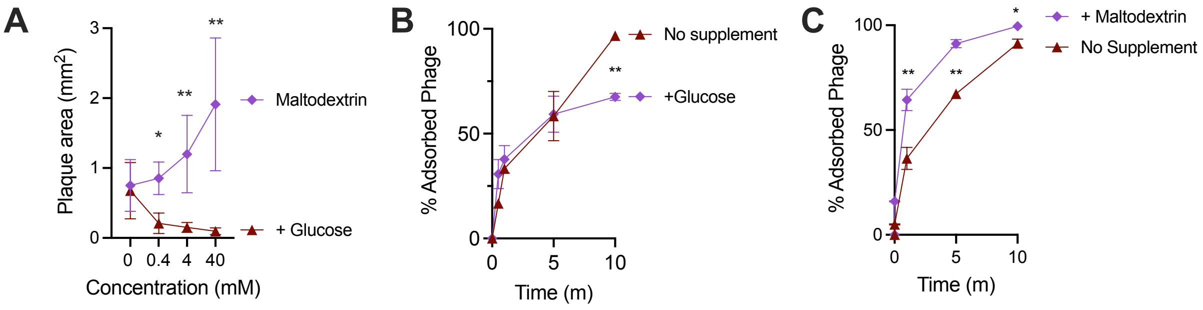

Figure 4: Exogenous sugars modulate phage adsorption to *E. coli* NC101. *E. coli* NC101 was grown in diluted (10%vol/vol) LB broth or agar supplemented with the indicated carbon sources at 37°C. **(A)** RSB03 plaque areas on NC101 lawns were measured after 18 hours. N = at least 30 plaques per condition. Statistical

comparisons between glucose and maltodextrin at the same concentrations (0.4, 4, and 40 mM) were performed using unpaired Student's t tests; *P<0.05, **P<0.01. **(B and C)** The percentage of adsorbed RSB03 virions were measured in cultures supplemented with 40 mM of the indicated sugar at the indicated times post infection with an initial MOI of one. Data are the mean ± SEM of three experiments, *P<0.05, **P<0.01 by Student's t test.

Figure S6, there is a PDB crystal structure of LamB: 1MAL. I recommend the authors utilize this rather than the AF predicted structure and cite the paper PMID 7824948.

This change was made and the paper cited.

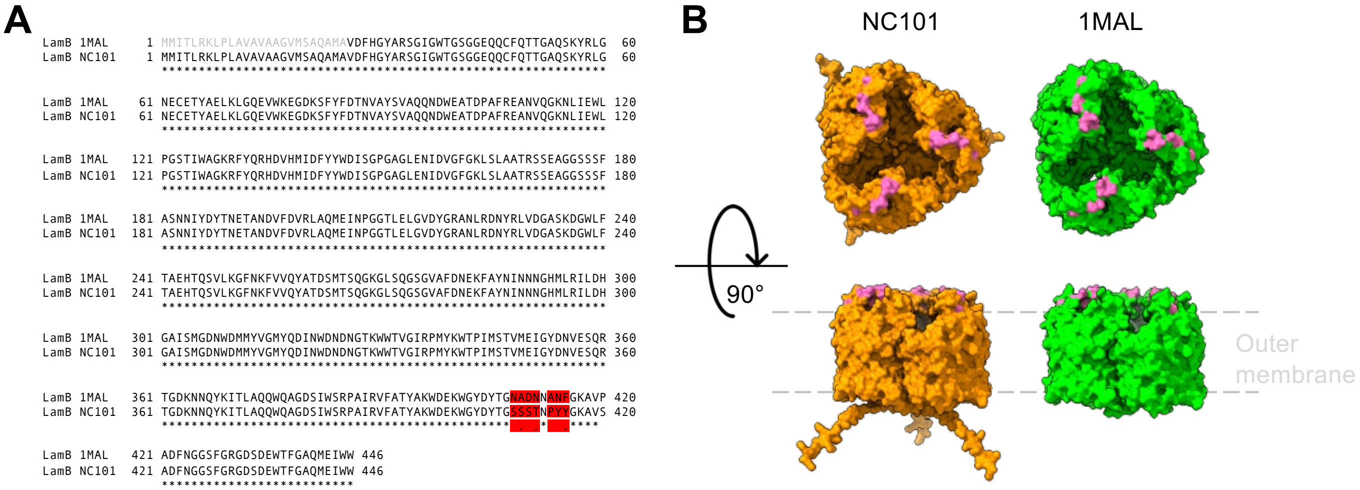

Figure S10. Comparison of LamB sequences and structures from *E. coli* NC101 and *E. coli* K-12 (1MAL). (A) LamB protein sequences were aligned using Clustal. Amino-acid differences between the two sequences are highlighted in red. The N-terminal signal peptide and periplasmic residues (1–25) shown in gray were omitted from the solved LamB crystal structure (PDB 1MAL). **(B)** Predicted LamB trimers from *E. coli* NC101 (AlphaFold model, orange) and the experimentally determined LamB structure (PDB 1MAL, green) are displayed as surface renderings. Residues that differ between NC101 and 1MAL LamB are shown in hot pink.

There is no statement in the methods indicating that testing was performed to confirm germfree status prior to gavage of germfree mice with E. coli. I assume this was done but recommend stating for completeness.

This change was made.

Reviewer #3: In this manuscript, Brzozowski and colleagues focus on prophage infection of adherent-invasive E. coli (AIEC), NC101. They identify a lambda-like prophage, NC-SV, that confer protection against two lytic phages isolated from the headwaters of the Missoula, Montana wastewater treatment plant (!), RSB01 and RSB03, in vitro. This was confirmed for RSB03 in vivo in mice. Comparison of the transcriptomes of E. coli NC101 and NC101ΔNC-SV 10 min post-infection identified over 200 genes that are differentially regulated. Genes associated with carbohydrate transport, amino acid metabolism and central carbon metabolism were over-represented. Based on LamB, a maltodextrin transporter, being the receptor for numerous coliphages the authors hypothesized that NC-SV is regulating LamB expression on the outer membrane to reduce phage adsorption. The study also identified a NC-SV

encoded small RNA that regulates more than 80 E. coli genes, including maltodextrin transport genes. Overall, the manuscript is nicely written, easy to understand and with a logical flow. The figures nicely represent the experimental content. I also feel that the bulk of the conclusions were supported by the data. Overall, it is a solid and impactful study. I provide some suggestions below on how to bolster the robustness of the LamB-phage infection angle of the manuscript and points in the text that require clarification.

Part II – Major Issues: Key Experiments Required for Acceptance
Please use this section to detail the key new experiments or modifications of existing experiments that should be absolutely required to validate study conclusions.

Reviewer #1:

1. Because the NC-SV prophage could encode virulence or transmission factors, it is premature to conclude that sensitivity to predation by RSB03 is why the NC-SV deletion strain has a different bacterial load and dissemination in mice compared to the parent strain NC101. You must compare loads and dissemination for these two strains in mice in the absence of RSB03 phage.

We thank the reviewer for this important comment. To directly address the concern, we compared bacterial loads and dissemination of NC101 and NC101$^{\Delta NC\text{-}SV}$ in the absence of RSB03 phage across multiple contexts:

- SPF mice lacking baseline Enterobacteriaceae (Fig. S5A–B)
- Gnotobiotic mice with a defined community including the closely related E. coli strain Mt1B1 (Fig. S5C–D)
- Monoassociated gnotobiotic mice (Fig. S7A–C)

Across these experiments, the only differences in intestinal colonization between strains were observed in the setting of competitive colonization in gnotobiotic mice containing Mt1B1, which itself encodes an identical NC-SV prophage. In SPF and monoassociated gnotobiotic mice, intestinal colonization was not significantly different between NC101 and NC101ΔNC-SV. With respect to extraintestinal dissemination, no statistically significant differences were observed in either SPF or monoassociated gnotobiotic mice. Interestingly, if anything, NC101ΔNC-SV trended toward increased CFU detection in the liver and spleen of SPF mice, opposite to the pattern observed in our gnotobiotic studies following RSB03 treatment. These new results are included in the revised manuscript as Fig. S5 and Fig. S7 (see Results, lines 175–198).

2. The paper seems incomplete without knowing how the NC-SV-encoded sRNA downregulates LamB. Did you computationally search for putative targets of the sRNA?

We thank the reviewer for raising this point. To explore potential sRNA binding targets, we searched the *E. coli* NC101 genome for homologous sequences to the sRNA using BLASTn. Even with a relaxed E-value threshold of 0.5, no significant matches were found. This suggests that the sRNA may not act through direct base pairing with a complementary RNA target. As noted in the manuscript (lines 420–423), one possibility is that the sRNA functions through interaction with an RNA-binding proteins (e.g., Hfq, CsrA) rather than by direct antisense regulation.

3. Although there are differences in plaque area, there is no in vitro data regarding phage EOP? Wouldn't you expect a change in EOP due to change in surface receptor expression?

Thank you for this suggestion. In response, we measured phage titers (PFUs/mL) of each RSB phage on NC101 and the three prophage mutants. Our PFU measurements showed no significant differences for RSB01, RSB02, or RSB03 across strains. The only exception was RSB04 on NC101$^{\Delta NC\text{-}MV}$, which displayed a modest but statistically significant increase in PFUs compared to NC101 ($P = 0.048$; see Fig S3). Thus, while plaque area differences were observed, overall phage titers were not reduced for the RSB phages on the prophage mutant backgrounds *in vitro*. This is now discussed on lines 140-148 of the revised manuscript.

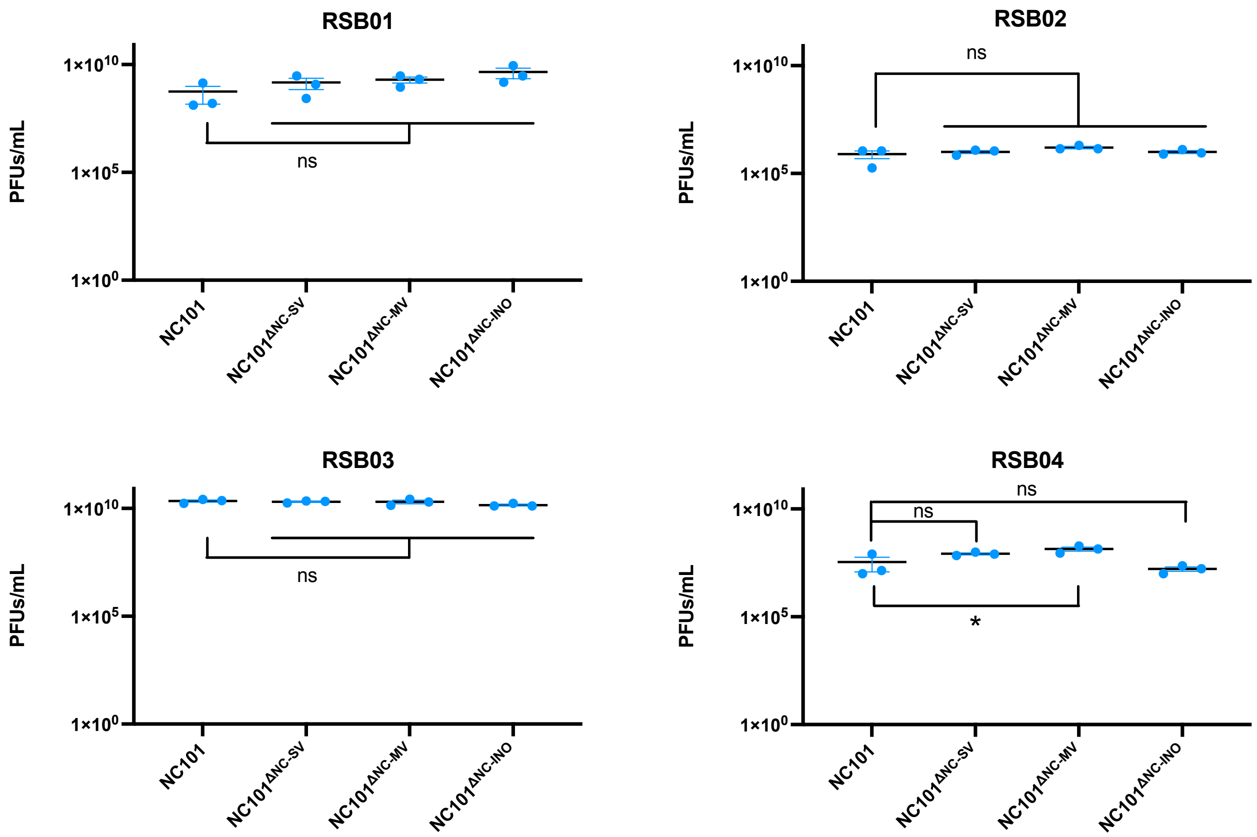

Figure S3. Phage titers of RSB01–RSB04 on NC101 and prophage-deletion mutants. *E. coli* strain NC101 and its isogenic prophage deletion derivatives (NC101$^{\Delta NC\text{-}SV}$, NC101$^{\Delta NC\text{-}MV}$, and NC101$^{\Delta NC\text{-}Ino}$) were infected with wastewater-derived lytic phages RSB01–RSB04. Phage titers were quantified as plaque-forming units per milliliter (PFU/mL) from three independent experiments (n = 3 biological replicates, individual data points shown, mean ± SEM). Statistical significance was determined by unpaired Student's t test comparing PFUs on each mutant strain to wild-type NC101; ns, not significant; $^*P < 0.05$.

Reviewer #2: (No Response)

Reviewer #3:

(1) The maltodextrin and glucose addition experiments support that the phage receptor is regulated by carbohydrates. I suspect that many genes encoding carbohydrate transporters are regulated by these sugars, however. If MalB is the actual receptor for RSB103 (and RSB101), then an E. coli NC101ΔmalB strain should be resistant to infection by these lytic phages. This experiment is key to confirming that these phages bind to MalB on the bacterial surface.

Thank you for bringing up this important point. We constructed an *E. coli* NC101 Δ*lamB* mutant and an NC101$^{\Delta NC\text{-}SV}$Δ*lamB* double mutant and evaluated their susceptibility to phage RSB03. As shown in the new Figure 3C–D, deletion of *lamB* did not affect RSB03 phage titers in liquid culture, indicating that LamB is not required for infection. However, plaque size was significantly reduced in the NC101$^{\Delta NC\text{-}SV}$Δ*lamB* strain relative to NC101$^{\Delta NC\text{-}SV}$, suggesting that LamB contributes to plaque expansion, possibly through effects on outer-membrane permeability or carbohydrate transport/metabolism rather than serving as the primary receptor. These results demonstrate that LamB modulates, but is not essential for, RSB03 propagation, which is consistent with our RNA-seq data showing *lamB* downregulation in NC-SV–infected cells.

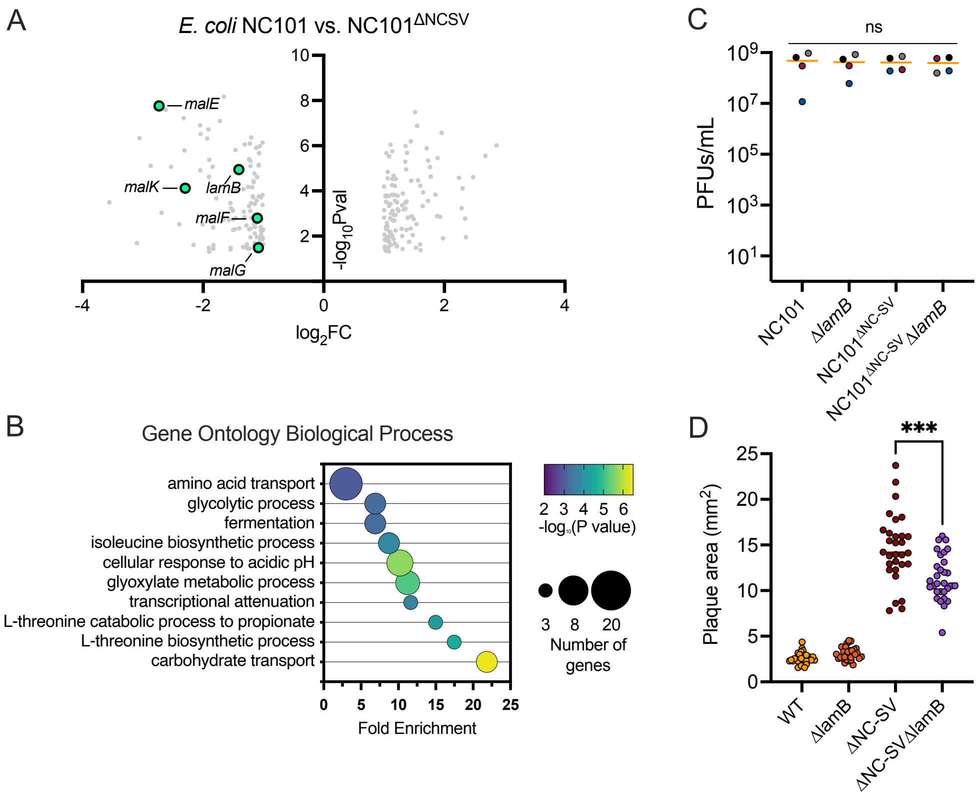

Figure 3. The NC-SV prophage transcriptionally regulates maltodextrin transport and carbon metabolism in response to phage infection. RNAseq was performed on mid-log NC101 or NC101$^{\Delta NC\text{-}SV}$ cells growing in LB broth 10 minutes post infection with phage RSB03 (MOI=1). **(A)** Volcano plot showing the 235 differentially expressed genes in NC101 compared to NC101$^{\Delta NC\text{-}SV}$ with a ±2-fold change and P≤0.05. Data are representative of quadruplicate experiments. **(B)** Gene enrichment analysis was performed on significantly differentially regulated genes shown in panel A. **(C)** Plaque area of RSB03 on lawns of NC101, Δ*lamB*, NC101$^{\Delta NC\text{-}SV}$, or NC101$^{\Delta NC\text{-}SV}$Δ*lamB*. Plaques area was measured using Image J after 18 h of growth; each point represents one plaque, data are the mean +/-SEM of three independent experiments, ****P < 0.0001, ns = not significant. **(D)** Phage titers (PFU/mL) recovered from RSB03 infections of the indicated strains in broth culture. Data are the mean +/-SEM of three independent experiments.

(2) The altered production of MalB (i.e. at the protein level) should be confirmed for the ΔNC-SV strain, also for the ΔNC-SV sRNA strain compared to empty vector. Whole cell lysates would be great, surface expression would be even better.

We appreciate this helpful suggestion. While we did not perform protein-level analyses of LamB expression in this study, our RNA-seq datasets provide strong transcriptional evidence that both the NC-

SV prophage and its encoded sRNA, *svsR*, downregulate *lamB* and other genes in the maltodextrin transport operon. Specifically, in *E. coli* NC101ΔNC-SV, the presence of NC-SV resulted in reduced expression of *lamB*, *malE*, *malF*, *malG*, and *malK* (Fig. 3A–B). Expression of *svsR* alone in the NC101ΔNC-SV background reproduced this transcriptional pattern (Fig. 6F–G), downregulating the same set of maltodextrin transport genes as well as additional carbohydrate metabolism pathways, including glycerol and N-acetylglucosamine utilization. These results demonstrate that the prophage and its sRNA independently alter the transcriptional program of *E. coli* in ways that likely affect surface physiology.

Consistent with these findings, our functional assays revealed that deletion of *lamB* does not abolish RSB03 infection but significantly reduces plaque size in the NC101ΔNC-SV background (Fig. 3C–D). Together, these data support a model in which NC-SV and *svsR* act at the transcriptional level to remodel carbohydrate transport and outer-membrane composition, thereby modulating phage adsorption and spread. We agree that direct confirmation of LamB abundance and/or surface localization in ΔNC-SV and ΔNC-SV + *svsR* strains would be a valuable next step, and future studies will include quantitative proteomics or surface biotinylation assays to address this point.

Part III – Minor Issues: Editorial and Data Presentation Modifications

Please use this section for editorial suggestions as well as relatively minor modifications of existing data that would enhance clarity.

Reviewer #1:

1. Line 100: Where is the key for the color-coded functional modules? Same for Fig S2.

We apologize for the confusion, the color coding of the ORFs is arbitrary and we have revised the legends for these figures for accuracy.

2. Line 122: Better to call them virulent phages throughout rather than lytic phages. Temperate phages have a lytic cycle, after all.

This change was made.

3. Figure 1:
A. Is DNC-MV plaque size also a bit smaller?

Fig 1B shows that ΔNC-MV plaque size is not significantly different compared to wt.

B. Panel D: move legend to side as it appears to be data points.

This change was made.

C. Max adsorption is reached by 10 min, so how much does this difference matter at 5 min?

Thank you for this insightful comment. While maximum adsorption is indeed reached by 10 minutes, we note that the reduced adsorption of RSB03 to NC101 compared to NC101ΔNC-SV is already apparent at the earlier 5-minute timepoint. This difference in adsorption kinetics correlates with the smaller plaque

sizes observed on NC101 lawns. Importantly, the adsorption assay was performed in liquid culture, whereas plaque formation occurs on solid surfaces where diffusion and adsorption dynamics may differ. Taken together, these results support the interpretation that the NC-SV prophage modifies a bacterial surface receptor to reduce virion adsorption, which in turn contributes to smaller plaque sizes.

D. Panel E: It is unclear what is being shown here. Is the final panel showing a cell with a burst region of the cell envelope? The figure legend should say something to help the reader.

We have clarified the legend to better describe what is shown in Panel E. The legend now reads:

"Representative transmission electron microscopy images of negatively stained NC101 and NC101ΔNC-SV cells 60 minutes post-infection with phage RSB03 (MOI 1). The final panel depicts an NC101ΔNC-SV cell undergoing phage-induced lysis, with loss of cell envelope integrity. Scale bars represent 500 nm or 50 nm in the inset."

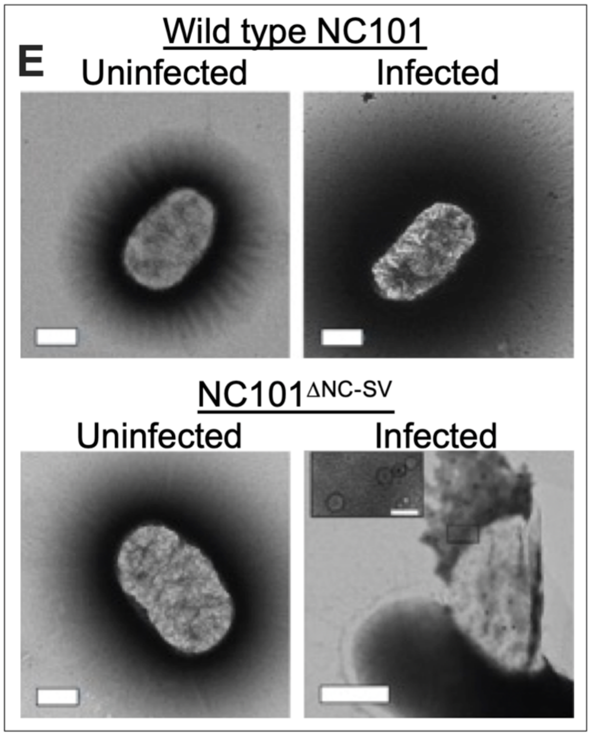

E. The conclusion that the cell surface receptor makes them more prone to lysis is one possibility. Couldn't it also be that NC-SV encodes for a protein that triggers phage defense systems that recognize RSB01/03? If the cell surface receptor plays a role in membrane integrity, shouldn't you show this through other methods such permeability to ethidium bromide or the like?

We appreciate the reviewer's thoughtful suggestion. It is true that prophages can encode a variety of defense systems, and we cannot rule out that NC-SV may harbor other genes that influence susceptibility to RSB01/03. However, our genetic and phenotypic evidence directly support the model that NC-SV modulates a bacterial surface receptor. Specifically, differences in adsorption kinetics and plaque sizes between NC101 and NC101ΔNC-SV are consistent with receptor availability being altered, and these observations align with our RNA-seq results showing regulation of carbohydrate transport genes linked to receptor function. While we did not perform complementary assays such as dye uptake

to directly probe membrane integrity, our data collectively support a receptor-based mechanism as the most parsimonious explanation. We agree that exploring additional potential prophage-encoded defense systems and assays of membrane permeability would be valuable directions for future work.

4. Figure 2:
A. The quality of the figure is bad. Too pixelated.

High resolution tiff images were submitted with the revised manuscript.

B. Panels G-I: there is no description in the legend to indicated what A, B, C or D mean above each bar graph (same comment for figure 5S).

We appreciate the reviewer drawing our attention to this point. We have revised the legends for Figures 2 and S5 to clarify the lettering above the bar graphs. Specifically, we now indicate that different letters (A, B, C, D) denote groups that are statistically different from one another, while bars sharing the same letter are not significantly different.

5. Figure S6: Should you consider that inability of lambda phage to infect NC101 might also be due to phage super immunity that's not related to the phage receptor, but suppression of phage cycle?

We agree that the inability of lambda phage to infect NC101 could reflect superinfection immunity or other prophage-encoded mechanisms that suppress the phage replication cycle, in addition to the receptor-based explanations discussed in the manuscript. However, lambda also failed to infect the NC101ΔNC-SV strain, indicating that NC-SV itself does not confer a lambda-specific superinfection exclusion phenotype. It remains possible that one or both of the other prophages in the NC101 genome (NC-Ino or NC-MV) could mediate superinfection exclusion or otherwise interfere with lambda replication. We now note this possibility (lines 259-262) as an alternative explanation for the observed lambda resistance phenotype:

"This outcome might be due to mutations in the NC101 LamB protein that are localized to its extracellular region [51]—the primary site of phage interaction [52] (Fig S10). Alternatively, it is possible that the other prophages in the NC101 genome (NC-MV or NC-Ino) provide protection through superinfection exclusion or other mechanisms that suppress lambda replication, which could contribute to the observed lambda resistance in NC101 independent of LamB sequence differences."

6. Figure S7: Why isn't RSB01 adsorption being impacted by glucose supplementation? Does it have an alternative receptor?

We appreciate this thoughtful question. One explanation is that although RSB01 and RSB03 are related Veterinaerplatzviruses, they may not engage LamB in exactly the same way. It is possible that RSB01 can utilize LamB more efficiently under glucose-repressed conditions or as the reviewer suggests, that it has flexibility to engage an alternative or secondary receptor, which would buffer it from changes in LamB abundance. At present, our adsorption data suggest RSB01 is partially but not exclusively dependent on LamB, while RSB03 appears more directly influenced by LamB regulation. We now mention this possibility in the Discussion as an alternative explanation for the differential effects of glucose on these two phages on lines 274-279:

“Interestingly, while RSB01 and RSB03 are related phages (**Fig S2**), RSB01 adsorption was less affected by glucose supplementation than RSB03 (**Fig 4C; Fig S11**). This difference may reflect variation in how each phage engages LamB or the ability of RSB01 to utilize an alternative or secondary receptor under certain conditions.”

7. Figure 6A: there’s a pretty big difference growthwise in empty vector vs svsR expressing cells. Why is this?

We agree that the empty-vector control does not reach the same final density as the strain expressing svsR in the absence of phage (Fig. 6A). The effect emerges late in growth; the early exponential slopes are similar. One explanation is that svsR down-regulates LamB and other maltodextrin transport genes (Fig. 6F), reducing outer-membrane protein production and thereby slightly lowering biosynthetic burden, which could raise the final OD in rich medium. The empty-vector strain lacks this regulatory effect. Regardless, this small growth offset does not alter our central findings that svsR decreases LamB-dependent adsorption (Fig. 6E) and reduces plaque size (Fig. 6C–D). We now note this growth phenotype on lines 330-338:

“We next examined whether expression of the prophage-encoded sRNA svsR could reproduce the protective effects of the NC-SV prophage. In the absence of phage, svsR expression had little effect on exponential growth but produced a modest increase in final culture density compared to the empty-vector control (Fig. 6A). Upon RSB03 infection, svsR reduced bacterial lysis relative to the control strain (Fig. 6B). On lawns, svsR-expressing cells produced visibly smaller plaques (Fig. 6C) with an average area roughly half that of the empty-vector control (Fig. 6D). Consistent with reduced phage propagation, svsR significantly decreased RSB03 adsorption to NC101$^{\Delta NC\text{-}SV}$ cells (Fig. 6E).”

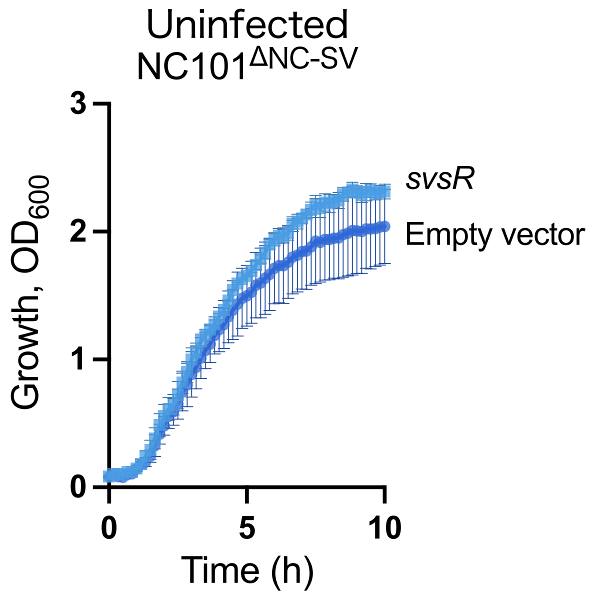

8. Figure 6: Although visually it appears svsR insertion behaves pretty much as WT from prior figures, it would be beneficial to compare it to WT NC101 to show if expression of svsR is sufficient to restore WT plaque area reduction and decrease in phage adsorption.

Thank you for the comment. We have addressed this by including in the results section the following: lines 338-340: “Notably, the phenotypes observed upon svsR expression in NC101$^{\Delta NC\text{-}SV}$ closely resemble those of WT NC101 reported in Fig 1: both exhibit smaller plaques and reduced phage adsorption compared to NC101$^{\Delta NC\text{-}SV}$.”

9. Material and methods: For the plaque assay, you don't say how many cells were used or if they are in top agar, etc. Instead there is information regarding the mouse experiment.

We now include more details on the plaque assay method: Phage plaque assays were performed using a standard soft-agar overlay method. Briefly, the indicated bacterial strains were grown overnight in LB medium at 37 °C with shaking. The following day, cultures were diluted (1:100) into fresh LB and grown to mid-log phase (OD600 ≈ 0.3–0.5). For each dilution of phage sample, 0.1 mL of phage suspension (serially diluted in phosphate-buffered saline) was mixed with 0.3 mL of host bacteria in soft agar (3 mL of molten soft agar, 0.6–0.7% agar, maintained at ~50 °C). After gently mixing, the soft agar suspension was overlaid onto prewarmed LB agar plates (1.5% agar base). Plates were allowed to solidify at room temperature, then incubated inverted at 37 °C for 18 hours. Discrete plaques were visualized and imaged. Individual plaques were outlined using ImageJ and plaque surface areas (in mm^2) were measured.

Reviewer #2: (No Response)

Reviewer #3: (3) Lines 148-152. "Phage RSB01 and RSB03 adsorption was significantly (P<0.01) higher in NC101ΔNC-SV compared to NC101 cells (Fig 1D, Fig S3H) while adsorption of phages RSB02 and RSB04 was not affected (Fig S3I and J), indicating that RSB02 and RSB04 use a different cell surface receptor than RSB01 and RSB03." It is surprising then that glucose reduces RSB03 adsorption to NC101 bacteria but minimal impact on RSB01. Please comment in the text as to why.

We appreciate this thoughtful question. One explanation is that although RSB01 and RSB03 are related Veterinaerplatzviruses, they may not engage LamB in exactly the same way. It is possible that RSB01 can utilize LamB more efficiently under glucose-repressed conditions or as the reviewer suggests, that it has flexibility to engage an alternative or secondary receptor, which would buffer it from changes in LamB abundance. At present, our adsorption data suggest RSB01 is partially but not exclusively dependent on LamB, while RSB03 appears more directly influenced by LamB regulation. We now mention this possibility in the Discussion as an alternative explanation for the differential effects of glucose on these two phages on lines 273-276:

"Interestingly, while RSB01 and RSB03 are related phages (**Fig S2**), RSB01 adsorption was less affected by glucose supplementation than RSB03 (**Fig 4C; Fig S11**). This difference may reflect variation in how each phage engages LamB or the ability of RSB01 to utilize an alternative or secondary receptor under certain conditions."

(4) It is not clear how prevalent the lambdoid prophage NC-SV is in E. coli strains. Is it restricted to adherent-invasive E. coli, for example? Please provide more background information about this aspect.

We thank the reviewer for raising this important point. To address it, we conducted a systematic survey of NC-SV–like sequences across E. coli genomes available in NCBI. Using BLASTn with a ≥10% genome coverage cutoff, we identified homologous prophage regions in 4,114 E. coli strains. By cross-referencing NCBI BioSample metadata, we found that NC-SV–like prophages are not restricted to adherent-invasive E. coli (AIEC). Instead, they are broadly distributed: 1,603 clinical isolates, 328 environmental isolates, and 109 laboratory strains carried NC-SV–like elements, while 2,137 accessions lacked sufficient metadata for classification. Pathotype assignments indicated wide representation, including STEC/EHEC (n = 297), ETEC (n = 147), UPEC/ExPEC (n = 113), EPEC (n = 4), and laboratory K-12 lineage strains (n = 99), in addition to AIEC (n = 2). The majority of genomes (n = 3,515) could not be confidently

assigned to a defined pathotype. Collectively, these results demonstrate that NC-SV–like prophages are widely disseminated across E. coli lineages and ecological sources, and are not restricted to AIEC. These data are mentioned on Lines 301-316.

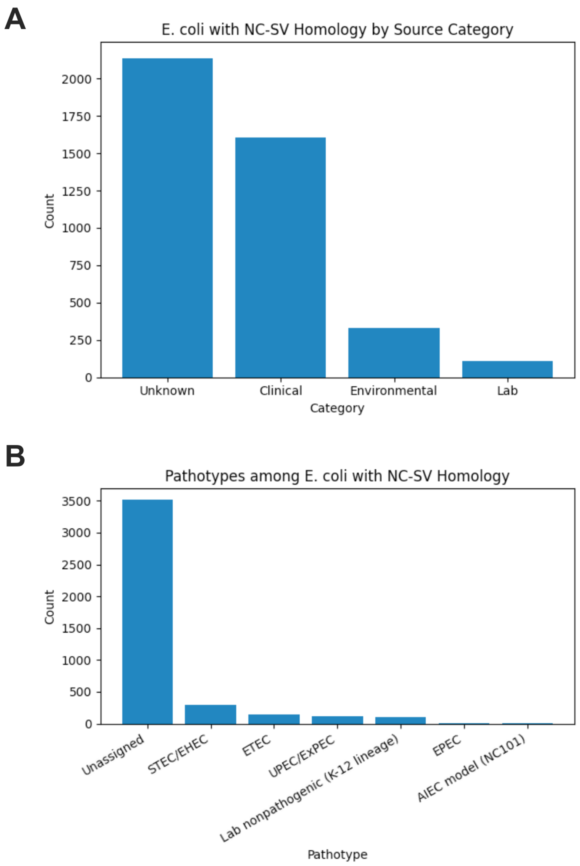

Figure S12. Distribution of *E. coli* genomes carrying NC-SV–like prophages by source and pathotype. (A) Source categories of *E. coli* assemblies containing NC-SV–like prophages, classified from NCBI BioSample metadata. Clinical isolates were the largest defined group (n = 1,603), followed by environmental (n = 328) and laboratory reference strains (n = 109). A substantial fraction (n = 2,137) lacked sufficient metadata for source assignment (*Unknown*). **(B)** Pathotypes of *E. coli* genomes with NC-SV–like prophages identified by BLASTn (≥ 10% genome coverage) across 4,114 *E. coli* assemblies. Most strains were unassigned (n = 3,515), but NC-SV–like prophages were also detected among multiple pathogenic subgroups, including STEC/EHEC (n = 297), ETEC (n = 147), UPEC/ExPEC (n = 113), EPEC (n = 4), as well as laboratory K-12 lineage strains (n = 99) and the AIEC model strain NC101 (n = 2).

(5) Line 89. “Neatly missing”. Odd choice of words. Suggest changing.

These are Sherwood Casjen’s words and we would like to preserve them.

(6) Table 1. It is difficult, if not impossible, to see the shading that indicates percent identity. Suggest changing graphics.

We thank the reviewer for this comment. We believe the concern may have been intended for **Fig. S1** rather than Table 1, as Fig. S1 displays clinker alignments with grayscale shading to indicate percent identity between NC-SV and λ. In this figure, the shading scale (0–100% identity) is shown directly below the alignments, and we find that differences in shading (e.g., light gray vs. black) are discernible. To

further assist readers, we have clarified the legend for Fig. S1 to explicitly note that shading intensity corresponds to the degree of sequence identity.

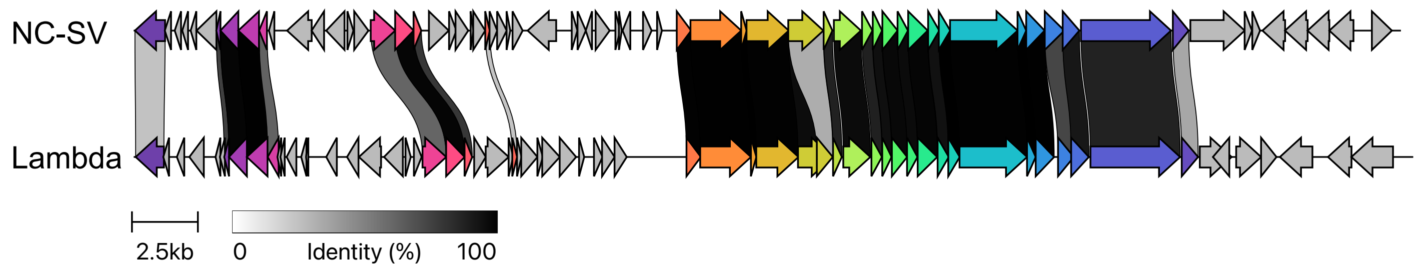

Figure S1. Comparative analysis of NC-SV and lambda prophages. Genomic alignment between the NC-SV prophage and phage Lambda. Arrows represent predicted open reading frames. Homologous genes are connected by ribbons shaded according to pairwise amino-acid identity (0–100%). The shading between genomes (where applicable) reflects nucleotide sequence identity across aligned genomic regions.

(7) A few y-axes have units listed as log(1+CFU/g), see Figure S4B and C, Figure 2G and H, Figure S5B and C for example. I believe this should be log10CFU/g.

The correct label is $\log_{10}$(1+CFU/g). This allows for plotting/statistical analysis of zero values (samples for which no colonies were counted) which do occur in the dataset. These have been updated in the relevant figures/figure legends.

(8) Figure S5 legend. I am confused about "Significantly different groups are indicated by compact letter display". I see there are small letters above the columns but I don't know what they mean.

We thank the reviewer for pointing out this source of confusion. We have revised the legend for Figure S5 to clarify the meaning of the compact letter display. Specifically, different letters above the bars (a, b, c, d) indicate groups that are statistically different from one another, while bars that share the same letter are not significantly different.

(9) Line 262. Suggest changing sentence to "This outcome might be due to seven amino acid mutations in the C-terminus of MalB that are localized to the extracellular region – the primary site of phage interaction". The alignment is much more striking than the existing verbiage.

This change was made.

(10) Line 342. Why was ≥10% genome coverage chosen? Please clarify in the text what this means?

We thank the reviewer for this question. For our prophage prevalence analysis, we defined NC-SV–like prophages as those sharing ≥10% genome coverage with the NC-SV reference sequence. This threshold was chosen to balance sensitivity and specificity: it is stringent enough to exclude spurious short matches but permissive enough to capture divergent prophages that still share conserved lambdoid gene modules. In practice, ≥10% coverage corresponded to tens of kilobases of aligned sequence, sufficient to encompass multiple conserved prophage structural and regulatory genes and reliably indicate the presence of related prophages. We have clarified this point in the revised text (lines 301-316).

(11) Line 621. Typo. Should be manufacturer’s.

This change was made.

(12) Transmission electron microscopy. I think there are some details missing. Were the bacteria embedded and sectioned?

We thank the reviewer for this comment. The TEM imaging was performed using whole-mount negative staining rather than embedding and sectioning. Cells were applied directly to carbon-coated grids and stained with uranyl acetate before imaging, and we have revised the Methods to clarify these details.
